# Escaping the Catastrophic Logic of Separation

**DOI:** 10.1089/heq.2022.29021.mtf

**Published:** 2023-01-20

**Authors:** Mindy Thompson Fullilove

**Affiliations:** The New School, New York, New York, USA.

**Keywords:** paradox of Apartheid, redlining, New Racism

## Abstract

In the philosophical system of American Apartheid, “race” is a fact, racial hierarchy a key corollary, and “separation of races” in status, rights, and geography a logical inference. The concept of racial hierarchy proved useful in the early colonial period in Virginia and elsewhere, first begun in the 1600s to keep indentured servants and enslaved people from joining together and overthrowing the plantation oligarchy. The discourse of separation has continued to be a key tactic for gaining and maintaining power, with profound harmful consequences for the nation. This article will explore three themes that are important for these times: (1) the ongoing “redlining system” that continues to ensnare us in a geography of apartheid; (2) the paradox of apartheid that “separation” binds the parts more firmly together; and (3) the “new racism” that attacks progress with the use of such farfetched ideas as the “replacement theory.” From these explorations, I propose ways in which TRUTH and RECONCILIATION can be mobilized to put us on a new path.

## Examining Premises of Separation

I open this essay by examining an obscure conflict in the pages of *HealthPAC*, an activist journal that set the tone for progressive health politics for many years. I find the conflict illuminating for two reasons. First, it involves the refusal to name a heinous and racist public policy—planned shrinkage—that is still denied and still in use. Second, it also involves the evasion of ecological analysis, which is central to addressing the catastrophes threatening the globe in 2022, as this is being written.

In summer 1991, the journal published an issue on “emerging health Apartheid” in the United States. In the introduction to the issue, the editors wrote,

…over the last decade we have witnessed some ominous developments: increasing numbers of mentally ill and homeless persons without shelter or care; skyrocketing numbers of young people in prisons; and untreated social epidemic of drug abuse, HIV-related disease, and interpersonal violence. All of these disproportionately affect people of color. Moreover, these conditions and events, which isolate and threaten the existence of our most impoverished and vulnerable communities, are met by growing indifference. There is an increasing unwillingness to provide even basic, humane levels of sustenance, particularly for those who can be characterized as less than ‘worthy’ of our concern. (p. 3)^1^

One of the articles in that issue was written by Drs. Rodrick Wallace and Deborah Wallace, who shared the findings of their ecological analysis of the effects of New York City's implementation of the policy of planned shrinkage.^2^ That disinvestment policy pulled fire services from neighborhoods deemed “bad,” triggering a fire storm that destroyed tens of thousands of housing units. The loss of housing forced massive numbers of people out of their neighborhoods.

The destruction of housing and the displacement of people set in motion a wide array of social, psychological, and health problems. The Wallaces noted,

The urban afflictions of homelessness, addiction, mental illness, AIDS, and sick children overwhelming our hospitals, and crime and violence overwhelming our neighborhoods and jails, are not separate and disparate problems. Rather, they are part of an interwoven pattern of urban ecological collapse and desertification whose remedy requires degrees of understanding and political will not often brought to public problems in the United States. (p. 13)

Rather than helping the readers understand the ecological concept of “urban desertification,” the editors' introduction to the article undermined the validity of the Wallaces' work, saying, “The *authors believe* that much of the deterioration in the South Bronx stemmed from a policy of ‘planned shrinkage’ on the part of the city… in order to displace the largely minority population and open areas for urban and industrial renewal. While *many may not accept* this theory of the origins of urban decay, this does not alter the usefulness of the Wallaces' analysis of its results” (p. 13; emphasis added).

I have to pause for a moment to reflect on the evocation of “belief” when commenting on scientific findings that had already been peer-reviewed and published in esteemed scientific journals. There are many methods for critiquing science, but calling it “belief” is not one of them. I am often approached by people who have read my articles or heard me talk who then tell me that they *believe* I am right. Aside from how annoying it is, the reason I bring it up here is that Americans do not understand the process of science and the role of “fact” in the assertion of findings. Furthermore, in that particular article, the Wallaces were not presenting a theory, as such, but rather a thorough analysis of an ecological process that had already been published in a scientific journal.^3^ The editors used “theory” in the same sense as “belief”—to discredit the science.

Which brings me to the key question and the reason for the dissection of this event: Why was the ecological analysis belittled in this manner?

To my reading, the editors of *HealthPAC* were making an effort to be anti-racist, but had not then understood the logic of Apartheid. They failed to grasp the significance of the Wallaces' challenges to three central assumptions of Apartheid. The first of these we might call the “always” premise, as in, “It's always been like that.” The passive voice is at the heart of the statement: nobody did it, it is just like that, probably because “*those* people” ____ {are dirty, don't take of their things, are dumb, etc.]. It is a remarkable and tenacious premise, one that defies rational analysis, as in the case I have noted here. The Wallaces were presenting scientific data that show that the *policy* led to ecological collapse. The editors made it clear that the critique of policy was just “belief” and not to be taken seriously. If it is true that it is always been like this, then policy did not do it.

Second, behind the wall of “always like this,” the powers-that-be have enacted Apartheid policies that created and then elaborated separation and stratification by race and class. Denial that such policies exist is characteristic of the Apartheid system, for example, denial that policies that enshrine police brutality have been enacted by legislatures and enshrined by the courts.

Third, the Wallaces were putting forward an ecological analysis, and ecology challenges another central premise of Apartheid, which is that separation is the inevitable and natural order of things. Ecology, the science of systems, is a science of interactions among the living and nonliving parts of the universe. It is inherently inclusive of all elements of a diverse world and, therefore, intolerable to the dominant Apartheid system.

I am picking on a progressive journal because its effort was to be anti-racist and inclusive. It is essential to understand that, in the struggle against Apartheid, we can easily fall into its traps. The system is inscribed at every level of scale, and that includes in our own thinking. A young black person who says, “I don't see someone who looks like me in [that group]” is struggling with both the exclusion they resist, as well as the “truth” that “looking like me” is defined by some characteristics made important by Apartheid. Such ideas are operating within the frame of the Apartheid premises of “always/separate” and have not shifted to an ecological analysis. If we are to make it through the challenges we face, we need to be highly self-aware.

In the following sections, I want to examine the ways in which an ecological analysis helps us see issues differently and opens a path to truth and reconciliation.

## Redlining

**FIG. 1.**
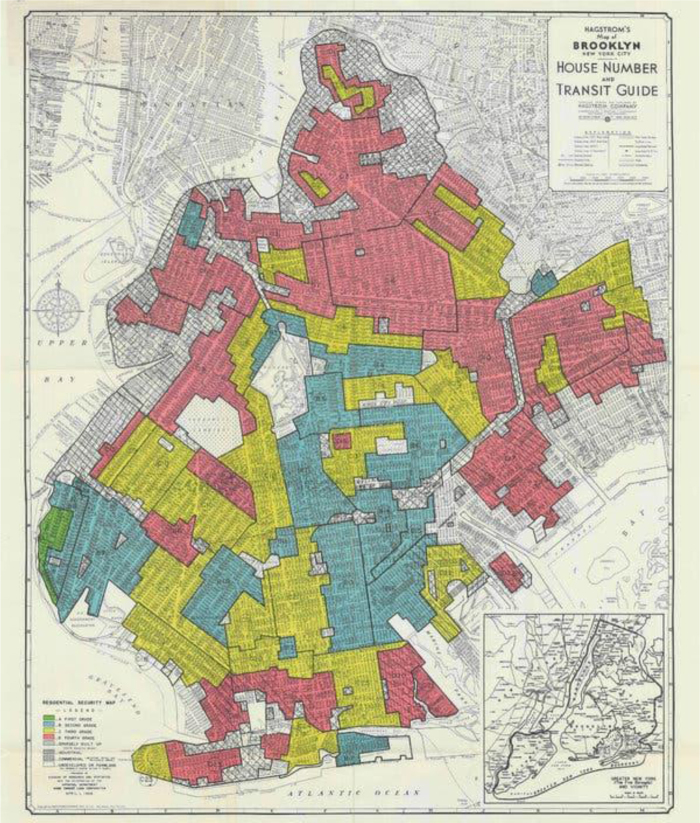
Redlining map of Brooklyn, NY.

In the 1930s the federal government carried out a project designed to rate areas of American city for their investment potential. The residential areas of major American cities were rated A, B, C, or D—basically reflecting age of buildings and presence of non-white people—and the maps were colored according to the area ranking. Although they are called “redlining” maps, these maps do not have any red lines on them. It was not a simple red line, but rather the color-coding of the whole city (Fig. 1).

In the past decade, dedicated scientists at several universities have made the 1930s redlining maps, and the surveys on which they were based, available on the internet. This has led to an explosion in studies of relationships between the geography those maps established and present urban conditions.^4–9^ More and more people have seen one or more of the maps and have explored the assumptions for grading that went into their making. Although much has changed in the intervening 85 years, researchers are finding striking correlations with tree cover, infant mortality, and other outcomes.

The Mapping Inequality website has a diagram that captures a different and equally important feature of the maps: the location of the more than 250 cities that were mapped and the proportion of each city that was coded for each color.^9^ On that diagram, we can see that American cities were largely coded yellow and red, meaning that investment should go elsewhere. The disinvestment in the American city, beginning with its minority neighborhoods, is one of the deep causes of change over the time since the codification of redlining. My own work on urban processes affecting African American communities after World War II amply documented that those neighborhoods were highly functional across many measures of well-being and social adaptability.^10^

The implementation of policies of urban renewal, followed by planned shrinkage, gentrification, the foreclosure crisis and emerging post-Covid eviction wave has displaced millions of African Americans and destroyed the social bonds that helped them both endure racism and poverty and remain advocates for democracy.^11^ The ills that the Wallaces were describing in their *HealthPAC* article—homelessness, addiction, infectious disease, infant and maternal mortality, and violence—have grown and acted synergistically to cause more ills. Thus, a description of a typical African American community 1950 is not the same as a description of such a community in 2020.

## The “Paradox of Apartheid”

After World War II, American capitalism entered a new period, which David Harvey has called “accumulation by dispossession.”^12^ Basically, the powers-that-be wrote policies, such as the urban renewal policy, that allowed “takings”—in that case, the taking of people's homes for so-called “progress.” Similarly and simultaneously, deindustrialization stripped the nation of its factories, sending them to countries with fewer labor and environmental protections, and leaving behind massive unemployment, brownfields, and desertification of former industrial centers.^13^ Such appropriations fed a massive inequality in wealth that has entrained a larger and larger proportion of the American population. The resulting stress has undermined the physical and mental health of the nation, the epidemics of obesity, depression, and substance use being three key symptoms of the processes imposed by Apartheid.

**FIG. 2.**
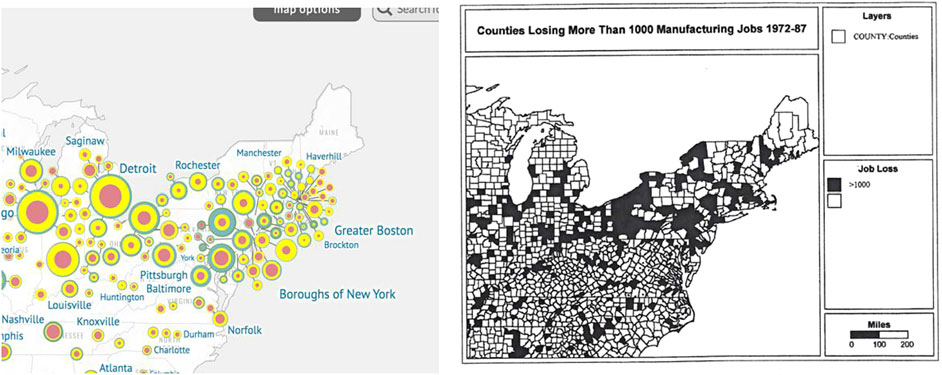
Left, redlined cities, right, deindustrialized cities.

I noted earlier the role of deindustrialization in the collapse of the American city. In Figure 2, we can see that the cities of the northeast were slated for disinvestment in the 1930s, which set up urban renewal in the 1950s. When we compare that map with the Wallaces' map of counties affected by deindustrialization, we see the whole industrial North—from the Atlantic to the Midwest—was involved in both processes. The pandemics of the 1980s and 1990s followed hard on the heels of these synergistic processes.

Furthermore, as the Wallaces point out, although the harms started in communities of people of color, they have worked their way up the social hierarchy, entraining us all, what the Wallaces have called the “paradox of Apartheid.” One of their findings most relevant to this essay is that, paradoxically, contrary to the supposed “separation” imposed by Apartheid, its policies of deindustrialization and urban desertification have actually bound the parts tightly together.

**FIG. 3.**
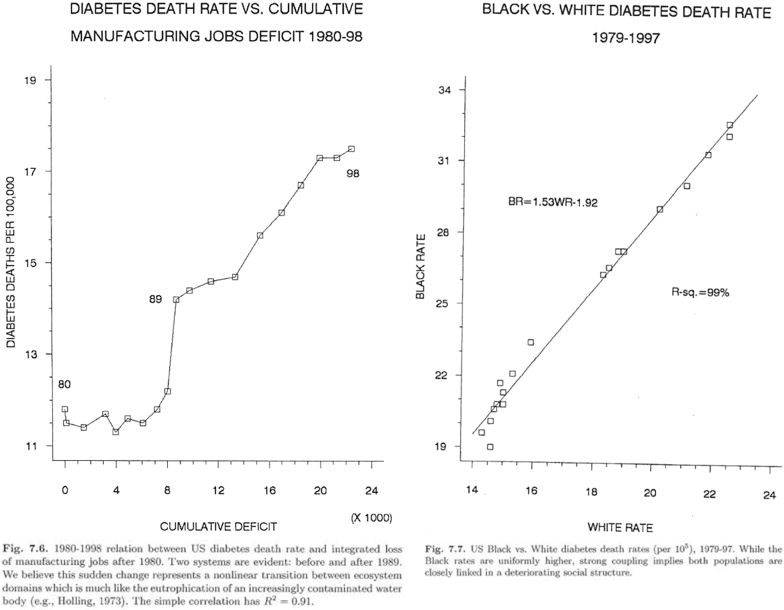
Disease and deindustrialization.

In his book, *Gene Expression and Its Discontents: The Social Production of Chronic Disease*, Rodrick Wallace illustrates this point with two illuminating Figures.^14^ In Figure 3, the graph on the left shows the U.S. diabetes death rate plotted against the loss of manufacturing jobs for the period 1980–1998. It is a graph striking for the sharp spike in deaths in 1988, which he suggests is similar to the kinds of tipping points seen in bodies of water faced with too much pollution. The second graph, on the right, compares the rates for blacks and whites for the period 1979–1997. If, indeed, apartheid had delivered on its premise of black suffering, white prosperity, the R-sq, a measure of similarity, would have been very low. Instead it is 99%, an astounding confirmation that instead of separation, we have a single stratified system. What happens to one, eventually happens to all.

## The “New Racism”

Surveys show a decline in racism in the United States for many decades. The General Social Survey, a national survey of Americans, has asked about race and racism since it began in 1972. In a *FiveThirtyEight* post on shifting attitudes toward race, author Anna-Maria Barry-Jester noted, “Since the 1970s, support for public and political forms of discrimination has shrunk significantly. While 31 percent of white Southerners favored segregated schools in 1972, by 1985, so few people shared that belief that the question was removed from the survey altogether.”^15^

But racism, the philosophy of Apartheid, has been able to adapt to the changing landscape. The hard core of people who had not shifted attitudes on race was one group that was available for such movements. As we noted in the section Paradox of Apartheid, the abandonment triggered by redlining and deindustrialization had affected more than the people of color at the bottom of the social hierarchy. For whites caught in these processes, the explanations offered by white supremacist groups created a path forward. This converged with neoliberalism and the general abandonment of social safety networks and widespread promotion of individual responsibility. Thus, what I am calling the “new racism” is rooted in the 1980s, but takes the public stage with the protests in Charlottesville in 2017.^16^

The Southern Poverty Law Center has followed the growth of the movements organized around racial hatred.^17^ Their virulent rhetoric, willingness to engage in violence, and disdain for the trappings of democracy have marked them as a profound threat. Although they have been labeled extremists, their influence through Fox News anchor Tucker Carlson and the rightwing of the Republican party makes it clear that they are not acting in isolation from larger political trends and processes in the United States. Their identification with historical sources of Nazism and Apartheid makes it clear that their goals are to preserve political power for “white” people.

At the heart of the success of this hard-right coalition is the success of racism in creating the illusion that the issues are black and white. The disruptions of repeated displacement and deindustrialization, the massive growth in inequality in wealth, and the threats posed by climate change get hidden behind the Big Lies such as the Great Replacement Theory. Given the general shift away from belief in racist stereotypes, racism has resorted to new kinds of fear-mongering. Identity politics, in general, by advancing the causes of select groups, may be said to be part of this general scenario of separation.

## No Magic Bullet

The inexorable struggle of African Americans for justice and equality has been fundamental to the shift in the collective consciousness in the nation away from racism. The Samuel DeWitt Proctor Conference, a convention of liberal black churches, noted in a statement on the 2022 overturning of Roe vs. Wade:

We will continue to encourage those who believe in freedom to stay the course. We will continue to remind them that not only has the entire existence of Black people in this country been tainted by racist and sexist laws, but also that our ancestors pushed against them. There was no government, no law enforcement agency… and no judicial system that protected them. They worked against injustice because they had to and because the God they worshipped told them that they must.^18^

The statement is helpful in framing the breadth of the struggle against racism and inequality that has been waged in this country. While doctors in the early 20th century had a moment of euphoria with the invention of antibiotics—which they dubbed “magic bullets” to fight germs—doctors at the beginning of the 21st century know that there are no magic bullets. The capacity of germs to evolve has meant they have developed ways to evade antibiotics. Our overenthusiasm for disease control with magic bullets may mean that in decades ahead this tool for fighting infection will be lost to us.

**FIG. 4.**
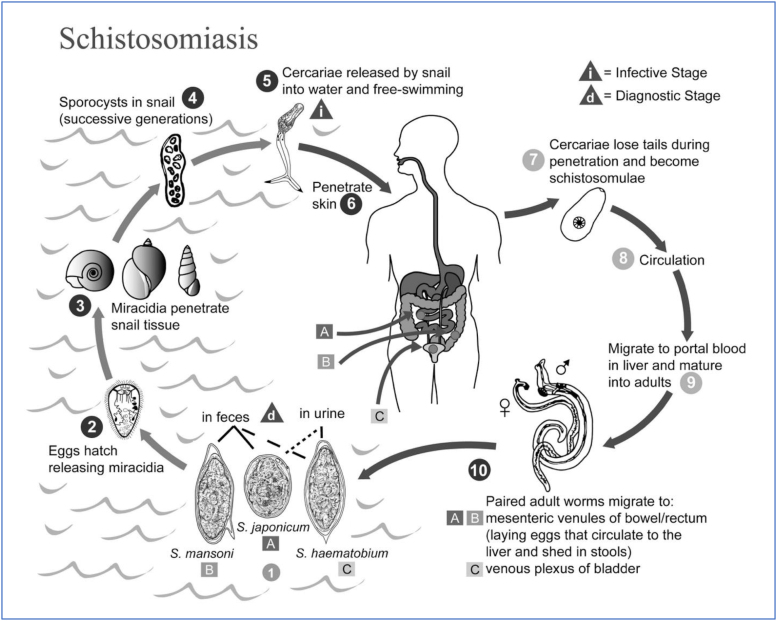
The lifecycle of schistosomiasis.

At the same time, the science of ecology has helped us understand that we can fight disease using “magic strategies”: sets of tools that address the problem at multiple levels of scale. One such example is that of schistosomiasis, a parasite that causes millions of infections a year leading to disability and death.^19^ The schistosome has a complicated life cycle, moving from water, to snails, to humans, and back to water. Multipronged approaches are essential for control of this problem. These include treatment of the infection, sanitation, protecting open bodies of water from urine and feces, and eliminating the snail hosts (Fig. 4).

Similarly, Drs. Deborah and Rodrick Wallace have argued that “magic strategies” are essential to managing complex social issues.^20^ These are strategies that address the problem at multiple levels of scale and through multiple systems. The problem of separation in the United States is certainly such a problem. It exists as a real geography, an ideology, and an illusion. These must be tackled at the same time, otherwise simple interventions simple strategies, such as other magic bullets, can be overrun by the people who wish to use racism to their advantage.

What, then, is the magic strategy that might be used to address separation?

**FIG. 5.**
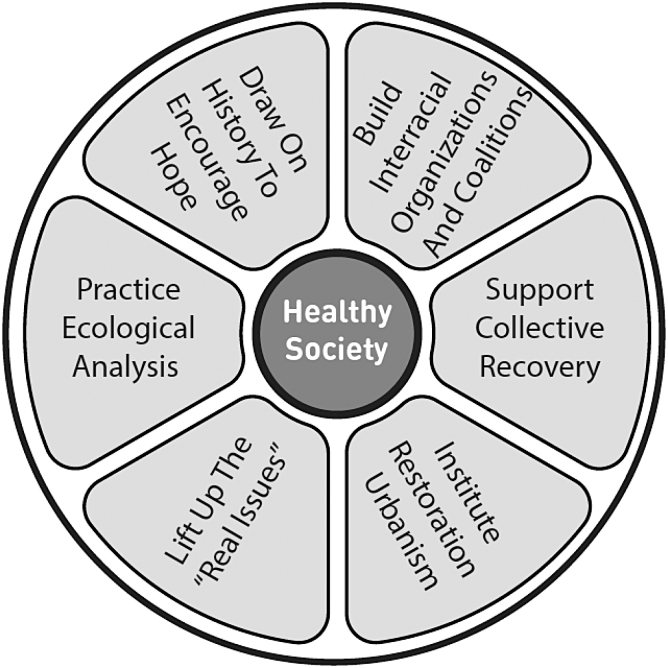
Magic strategy to end separation.

I want to suggest a magic strategy of six tactics (Fig. 5):

1. Practice ecological analysis: It is essential that ecological analysis become the standard in these complex times. Anti-racism has a natural affinity for ecology, which should be developed.
2. Lift up the “real issues”: It is impossible to make progress if we are not actually making progress. The real issues such as housing, wages, and climate change must be front and center all the time. Racism was invented to divide us and to hide the scam that is going on. Let us not forget what is behind the curtain.
3. Institute restoration urbanism: The American city has been fractured physically and socially as a result of the imposition of the geography of Apartheid. Restoration urbanism offers us a group of tools for repairing this fracture and making our cities whole.
4. Support collective recovery: The emotional tool of racism is everywhere around us, and its injuries need to healed. There are conversations to be had, songs to be sung, dances to be danced if we are to get past this traumatic history.
5. Build interracial organizations and coalitions: Identity politics have taken center stage in the United States in the past decades, but as long as these politics led to groups acting in isolation, they become part of the problem, not part of the solution.
6. Draw on history to encourage hope: It is said that Frederick Douglass was very discouraged at one point in 1847. Sojourner Truth said to him, “Frederick, is God dead?” Whether or not one believes in God, knowing those great leaders held on to end slavery can give us hope that our struggles will also prevail.
